# Sensitivity has Multiple Heterogeneity Problems: a Reply to Wallbridge

**DOI:** 10.1007/s11406-017-9873-5

**Published:** 2017-07-11

**Authors:** Guido Melchior

**Affiliations:** Department of Philosophy, University of Graz, Heinrichstrasse 26/5, 8010 Graz, Austria

**Keywords:** Sensitivity, Higher-level knowledge, Bootstrapping, Heterogeneity problem

## Abstract

In this paper, I defend the heterogeneity problem for sensitivity accounts of knowledge against an objection that has been recently proposed by Wallbridge in *Philosophia* (2016b). I argue in (Melchior in *Episteme*, *12*(4), 479–496, 2015) that sensitivity accounts of knowledge face a heterogeneity problem when it comes to higher-level knowledge about the truth of one’s own beliefs. Beliefs in weaker higher-level propositions are insensitive, but beliefs in stronger higher-level propositions are sensitive. The resulting picture that we can know the stronger propositions without being in a position to know the weaker propositions is too heterogeneous to be plausible. Wallbridge objects that there is no heterogeneity problem because beliefs in the weaker higher-level propositions are also sensitive. I argue against Wallbridge that the heterogeneity problem is not solved but only displaced. Only some beliefs in the weaker higher-level propositions are sensitive. I conclude that the heterogeneity problem is one of a family of instability problems that sensitivity accounts of knowledge face and that Wallbridge’s account raises a further problem of this kind.

## The Heterogeneity Problem

Nozick (1981) defines that a belief that *p* formed via method M is sensitive iff in the nearest possible worlds where *p* is false and where S uses M for determining whether *p* (or ¬*p*) is true, S does not believe via M that *p*. Nozick argues that sensitivity is necessary for knowing. I argue in (Melchior 2015) that sensitivity accounts of knowledge face a particular problem when it comes to higher-level knowledge. Beliefs that one does not falsely believe that *p* are insensitive if their underlying formal structure is ¬(B(*p*) ∧ ¬*p*). I label these beliefs B(*d*), since they have the formal structure of a disjunction. However, beliefs that one truly beliefs that *p*, with the underlying formal structure B(*p*) ∧ *p*, are sensitive if the belief that *p* is sensitive. Moreover, beliefs that one’s belief that *p* is not false with the formal structure B(*p*) ∧ ¬¬*p* are also sensitive if the belief that *p* is sensitive. I label these beliefs B(*c*) since they are beliefs in a conjunction. Notably, *c* is a stronger proposition than *d*. Thus, sensitivity accounts of knowledge have it that we are in a position to know the stronger proposition *c* without being in a position to know the weaker proposition *d*. This outcome is highly implausible. I labeled this problem the heterogeneity problem for sensitivity accounts.

This heterogeneity problem marks a further step in a discussion about sensitivity and higher-level knowledge. Sosa (1999) and Vogel (2000) first pointed out that beliefs in *d* are insensitive. Sosa argued that it is an implausible instance of closure failure that one can know that *p* without knowing that *d* since *p* is a stronger proposition than *d*. Vogel concludes more directly that sensitivity accounts of knowledge preclude us from having any higher-level knowledge since we cannot know that *d*. Becker (2006) and Salerno (2010) then argued against Vogel that we can have higher-level knowledge according to sensitivity accounts since beliefs in *c* can be sensitive. The heterogeneity problem arises if one combines the orthodox views that beliefs in *c* can be sensitive but that beliefs in *d* cannot. Wallbridge (2016a) opposes these orthodox views by arguing that even beliefs in *d* turn out to be sensitive if we properly take the belief forming method into account. Based on this line of argumentation, Wallbridge (2016b) continues to argue that sensitivity does not suffer from any heterogeneity problem. I will show that his objection is doomed to fail. Wallbridge is partly right since some beliefs in *d* are sensitive. However, I will argue that some other beliefs in *d* are insensitive. Thus, Wallbridge’s account does not solve the heterogeneity problem; rather, it raises an additional problem of instability for sensitivity accounts. Or so I will argue.

Before analyzing Wallbridge’s objection, let me clarify my overall position on sensitivity. Given the objections that I raise against sensitivity accounts of knowledge, one might think that I am a convinced critic of the sensitivity principle. On the contrary, I find it intuitively appealing and I think that it deserves a crucial role in epistemology. However, I also think that ‘knowledge’ is the wrong concept for applying it. In *​Knowing and Checking *(book manuscript), I argue that sensitivity is necessary for checking (and similar epistemic enterprises like testing), but not for knowing. Thus, sensitivity marks a crucial distinction between knowing and checking. To be more explicit, I suggest that S cannot check whether *p* is true via method M if, in the nearest possible worlds where *p* is false and where S uses M for determining whether *p* is true, M indicates that *p*. Moreover, the sensitivity account of checking that I elaborate in *Knowing and Checking *does not suffer from those kinds of problems that I pose for sensitivity accounts of knowledge. Thus, there is nothing wrong with sensitivity, only with sensitivity accounts of *knowledge*.

## Wallbridge’s Case and Alternative Cases

Wallbridge (2016a, b) argues against orthodoxy that higher-level beliefs in *d* can be sensitive. He does so by discussing Vogel’s Omar case:

**OMAR**

You see your long-time friend Omar, who is a perfectly decent and straightforward sort of person. Noticing his shiny white footwear, you say, “Nice shoes, Omar, are they new?” Omar replies, “Yes, I bought them yesterday.” You know that Omar has new shoes, and that you believe that Omar has new shoes. You also know, if you think about it, that you don’t falsely believe that Omar has new shoes. (Wallbridge 2016b)

Wallbridge argues that my belief that I do not falsely believe that Omar got new shoes is sensitive if we take the belief-forming method into account as Nozick suggests. We can assume that Omar is a sensitive informant about his shoes since Vogel claims that I know that Omar has new shoes which implies, on a sensitivity account of knowledge, that this belief is sensitive. Wallbridge argues that in the nearest possible worlds where I falsely believe that Omar has new shoes, I believe it on a different basis than Omar’s testimony, since Omar is a sensitive informant about his shoes. For example, I might believe it on the basis of Lila’s testimony who is a very unreliable informant about the footwear of her friends. Thus, in the actual world, I believe that Omar has new shoes based on Omar’s testimony but on a different basis in the nearest possible worlds where I falsely believe that Omar has new shoes. Crucially, my belief that I do not falsely believe that Omar has new shoes is sensitive if in the nearest possible worlds where I falsely believe that Omar has new shoes *and* where I use the same method as in the actual world, I do not believe via this method that I do not falsely believe that Omar has new shoes. This sensitivity condition is actually fulfilled because Omar is a sensitive informant about his shoes. In the nearest possible worlds where I falsely believe that Omar has new shoes and where I ask Omar whether he has new shoes, I come to believe that he does not have new shoes.

Wallbridge’s argumentation presumably goes through if we fill in the details as he suggests, i.e. if we assume that in the nearest possible worlds where I falsely believe that Omar has new shoes, I believe it on a different basis than I believe it in the actual world. But note that this crucially depends on how the details are filled in. Take the following alternative case:

**ROBINSON CRUSOE**

Robinson Crusoe and Freitag are the only inhabitants of a small island. They make their cloth out of palm leaves and lianas. One day, Freitag sees his long-time friend Robinson Crusoe, who is a perfectly decent and straightforward sort of person. Noticing his newly bound footwear, Freitag says, “Nice shoes, Robinson! Are they new?” Robinson replies, “Yes, I made them yesterday.”

Wallbridge’s interpretation of OMAR depends on the assumption that there are other methods available for believing that Omar has new shoes and that worlds where I falsely believe that Omar has new shoes via some other method are closer than the nearest possible worlds where I falsely believe via Omar’s testimony that he has new shoes. However, this is not the case in ROBINSON CRUSOE, where presumably there is no other method available, for example testimony from someone else, that could deliver that Robinson Crusoe has new shoes beside Robinson’s testimony. Robinson Crusoe might be a very decent person such that possible worlds where he falsely reports that he has new shoes are very remote. Nevertheless, any of these remote possible worlds is still closer than possible worlds where Freitag believes via a different method than Robinson’s testimony that Robinson has new shoes. Thus, in the nearest possible worlds where Freitag falsely believes that Robinson has new shoes, he believes it via Robinson’s testimony. But in this case, in the nearest possible worlds where Freitag falsely believes that Robinson has new shoes and uses the same method as in the actual world, he comes to believe that he does not falsely believe that Robinson has new shoes. Thus, Freitag’s belief that he does not falsely believe that Robinson has new shoes is *insensitive*. Take a second example:

**MORPHELIA**

Sigmund, Morphelia’s psychotherapist, asks her about her dreams last night. Morphelia is a perfectly decent and straightforward sort of person. Morphelia tells Sigmund that she dreamt that five green eagles picked her up and flew her into an orange classroom.

Even if Morphelia is a very decent and straightforward person, the nearest possible worlds where Sigmund falsely believes that Morphelia dreamt five green eagles picked her up and flew her into an orange classroom are such that he believes it via testimony from Morphelia. This is so because possible worlds where someone else makes reports to Sigmund about Morphelia’s dreams (and especially that Morphelia had this particular dream) are presumably modally even more remote than worlds where Morphelia made a false report about her dreams. Thus, in the nearest possible worlds where Sigmund falsely believes that Morphelia dreamt last night that five green eagles picked her up and flew her into an orange classroom and where he uses the same method as in the actual world, he will believe via this method that he does not falsely believe that Morphelia dreamt last night that five green eagles picked her up and flew her into an orange classroom. Thus, Sigmund’s belief is *insensitive*.

Freitag’s belief and Sigmund’s belief are both insensitive because possible worlds where they falsely believe the target propositions via the same method as in the actual world are closer than possible worlds where they falsely believe it via a different method. In ROBINSON, this is the case due to social isolation, *viz*. under normal circumstances propositions like ‘S has new shoes’ can typically be falsely believed via different sources than S’s testimony. In contrast, propositions about the content of S’s dreams are normally only believed via S’s testimony. In some cases, like OMAR, beliefs in *d* might be sensitive as Wallbridge suggests, but, in some other cases, beliefs in *d* are insensitive. Let us have a more systematic look at the issue. Whether beliefs in *d* are sensitive depends on whether the following Wallbridge-condition *W* is fulfilled:

**Wallbridge’s condition**

S’s belief that she does not falsely believe that *p* formed via method M is sensitive iff the following Wallbridge condition *W* is fulfilled. *W*: Possible worlds where S falsely believes that *p* via some other method M* are closer than the nearest possible worlds where S falsely believes that *p* via M.

S’s belief that *p* formed via M is sensitive iff in the nearest possible worlds where *p* is false and S uses M for determining whether *p*, S does not believe that *p* via M. *W* is fulfilled iff in the nearest possible worlds where S falsely believes that *p*, S does so via M*. Thus, sensitivity is a feature of beliefs formed via method M whereas condition *W* characterizes a relation between M and alternative methods M*.

A belief that *p* formed via M can be sensitive yet fail to fulfill condition *W*. If S’s belief that *p* via M is sensitive then there is a neighborhood of possible worlds where *p* is false and where S does not believe that *p* via M. If in this neighborhood there exists a method M* via which S falsely believes that *p*, then *W* is fulfilled, otherwise not. Whether there is such a method M* is contingent. Thus, the connection between sensitivity and condition *W* is contingent.

The orthodox view about sensitivity and higher-level beliefs has it that beliefs in *d* are insensitive. The heterogeneity problem is based on the assumption that beliefs in *d* are insensitive but that beliefs in stronger propositions *c* are sensitive (if the basic belief that *p* is sensitive). Wallbridge is presumably right that some beliefs in *d* are sensitive. He suggests that the heterogeneity problem vanishes because beliefs in *c* and in *d* can be sensitive. We saw that he is mistaken. Some beliefs in *d* can be sensitive but others not. Consequently, the heterogeneity problem has to be reformulated as follows. Beliefs in *c* are sensitive if the basic belief that *p* is sensitive. Some beliefs in the weaker proposition *d* are sensitive, some others are not.

Where does this leave us? Epistemically, there is no crucial difference between cases like OMAR, ROBINSON or MORPHELIA. Thus, I do not see why I should be epistemically in a better position concerning Omar’s shoes than Freitag is concerning Robinson’s shoes or Sigmund is concerning Morphelia’s dreams. Consequently, it is problematic to evaluate these cases differently and to claim that we know in OMAR but not in ROBINSON or MORPHELIA. Even if Wallbridge’s analysis is correct that some beliefs in *d* are sensitive, some other beliefs in *d* are insensitive. Thus, Wallbridge does not solve the heterogeneity problem, rather he merely displaces it.

## Conclusion

The heterogeneity problem arises because higher-level beliefs in higher-level proposition *d* are insensitive but higher-level beliefs in stronger higher-level propositions *c* are sensitive. Thus, according to sensitivity accounts of knowledge, we have stronger higher-level knowledge but lack weaker higher-level knowledge which is a highly unsatisfactory outcome. Importantly, the heterogeneity problem is only one instance of a family of problems for sensitivity accounts that point towards the fact that sensitivity is too unstable over propositions and methods for being a criterion for knowledge. Nozick (1981) already noted that beliefs in the denial of the skeptical hypothesis, ¬*sh*, are insensitive, but that beliefs in conjunctions of ¬*sh* and ordinary external world propositions, ¬*sh* ∧ *p*, are sensitive. For example, my belief that I am not a deceived brain in a vat (BIV) is insensitive because in the nearest possible worlds where I am a deceived BIV I believe that I am not a deceived BIV. However, my belief that there is a computer in front of me *and* that I am not a deceived BIV is sensitive. In the nearest possible worlds where this conjunction is false there is no computer in front of me and I am still not a deceived BIV. However, in these possible worlds, I do not believe this conjunction because I fail to believe the conjunct that there is a computer in front of me. Thus, sensitivity accounts of knowledge are committed to accept that we can know that ¬*sh* ∧ *p* although we are not in a position to know that ¬*sh*. This outcome is counterintuitive. In this case, sensitivity is too unstable with regard to closely related proposition ¬*sh* and ¬*sh* ∧ *p*. In this respect, this problem is closely related to the heterogeneity problem of knowing *c* but not knowing *d*.^1^

Wallbridge aims at showing that beliefs in the weaker propositions *d* are sensitive and that they can, therefore, constitute knowledge. If his point goes through for any belief in *d*, then the heterogeneity problem could be solved. However, it does not. In fact, his account raises an additional problem for sensitivity. If one grants his analysis of OMAR, then some beliefs in *d* are sensitive as he suggests, but some others are insensitive. This problem is a further instance of the instability problems to which the heterogeneity problem belongs. If Wallbridge’s analysis is correct, then one is forced to reformulate the heterogeneity problem but Wallbridge does not solve this problem, without raising a new problem of a similar kind. In fact, Wallbridge’s analysis offers further evidence that the instability problems are the crucial problems for sensitivity accounts of knowledge.

## Footnotes

1 I argue in (Melchior 2014a) that DeRose’s (1995, 2010) indirect sensitivity account also suffers from this problem. DeRose uses the sensitivity principle for explaining our intuitions about knowledge, although he does not directly build a sensitivity condition into the concept of knowledge. DeRose’s account fails because sensitivity cannot explain our intuitions about knowing that the skeptical hypotheses are false. Intuitively, if we do not know that ¬*sh*, then we also do not know that ¬*sh* ∧ *p*. However, DeRose’s indirect sensitivity account predicts that we think that we do not know that ¬*sh*, but think that we know that ¬*sh* ∧ *p*. Moreover, I argue in (Melchior 2014b) that sensitivity accounts also face a particular generality-problem when it comes to higher-level knowledge of *c*. One might object that the generality problem is everybody’s problem but other externalistic accounts such as safety or process reliabilism at least avoid this particular version of it. With the heterogeneity problem and the problem of knowing a conjunction without knowing one of its conjuncts, sensitivity is too unstable concerning the involved propositions. As for the generality problem, it is particularly unstable concerning the method in question.
